# Supplementary material for: Mapping the spatial transcriptomic signature of the hippocampus during memory consolidation
Source: Nat Commun. 2023 Sep 29;14:6100. doi: 10.1038/s41467-023-41715-7 (PMC10541893; doi:10.1038/s41467-023-41715-7)
Supplement: Supplementary file 1 — Supplementary Information [file 41467_2023_41715_MOESM1_ESM.pdf]

Supplementary Information for:

Mapping the spatial transcriptomic signature of the hippocampus during memory consolidation

Yann Vanrobaeys[1,2,3], Utsav Mukherjee[1,2,4], Lucy Langmack [1,2,5], Stacy E Beyer[1,2], Ethan Bahl [3,6], Li-Chun Lin [1,2] Jacob J Michaelson[2,6], Ted Abel[1,2*] and Snehajyoti Chatterjee[1,2*]

Affiliations:

[1] Department of Neuroscience and Pharmacology, Carver College of Medicine, University of Iowa, Iowa City, IA, USA

[2] Iowa Neuroscience Institute, University of Iowa, Iowa City, IA, USA

[3] Interdisciplinary Graduate Program in Genetics, University of Iowa, Iowa City, IA 52242, USA.

[4] Interdisciplinary Graduate Program in Neuroscience, University of Iowa, Iowa City, IA 52242, USA.

[5] Biochemistry and Molecular Biology Graduate Program, University of Iowa, Iowa City, IA, USA

[6] Department of Psychiatry, University of Iowa, Iowa City, IA, USA

*Corresponding authors:

ted-abel@uiowa.edu

snehajyoti-chatterjee@uiowa.edu

This PDF file includes Supplementary Figure and Supplementary Figure Legend 1-8.

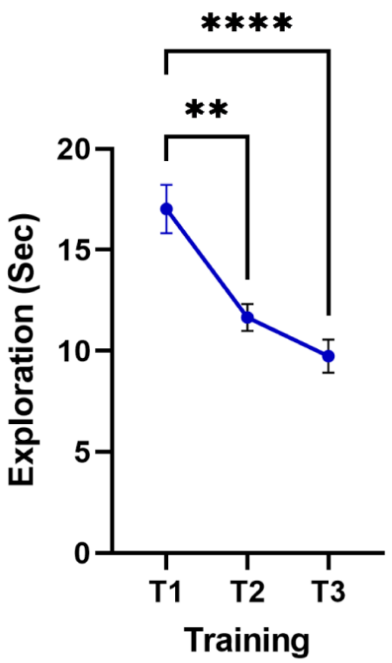

Supplementary Figure 1. Total exploration during training sessions. a. Exploration of the objects during the training sessions declined significantly. One way Anova: F (2, 18)=0.608, *P*<0.0001. Tukey's multiple comparison tests: **P= 0.0017 (T1 vs T2), and ****P <0.0001 (P= 0.00007243250228, T1 vs. T3). n=7. Error bars represent ± SEM.

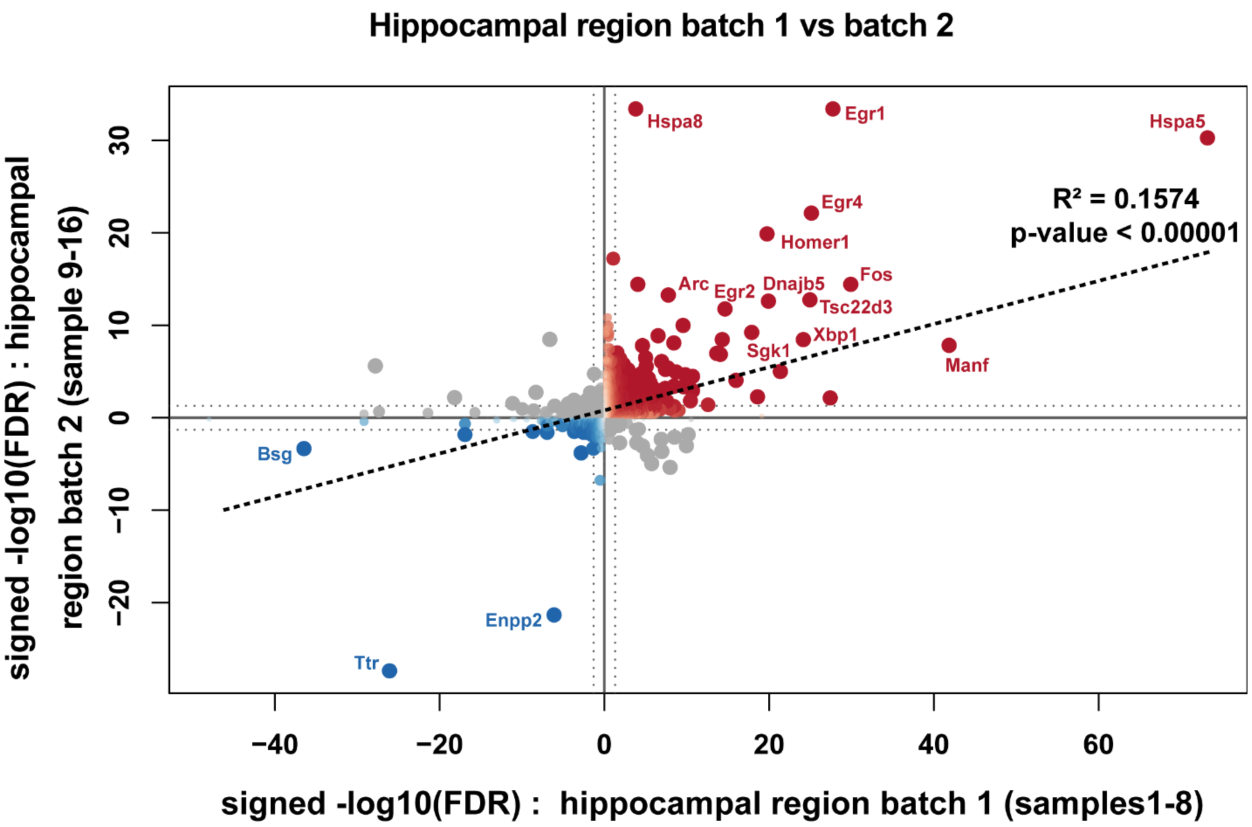

Supplementary Figure 2. Quadrant plot comparing the transcriptomic profiles between the two batches of spatial gene expression experiments. Correlation batch 1 (Bahl et al 2022, GEO GSE201610) vs batch 2 (current study).

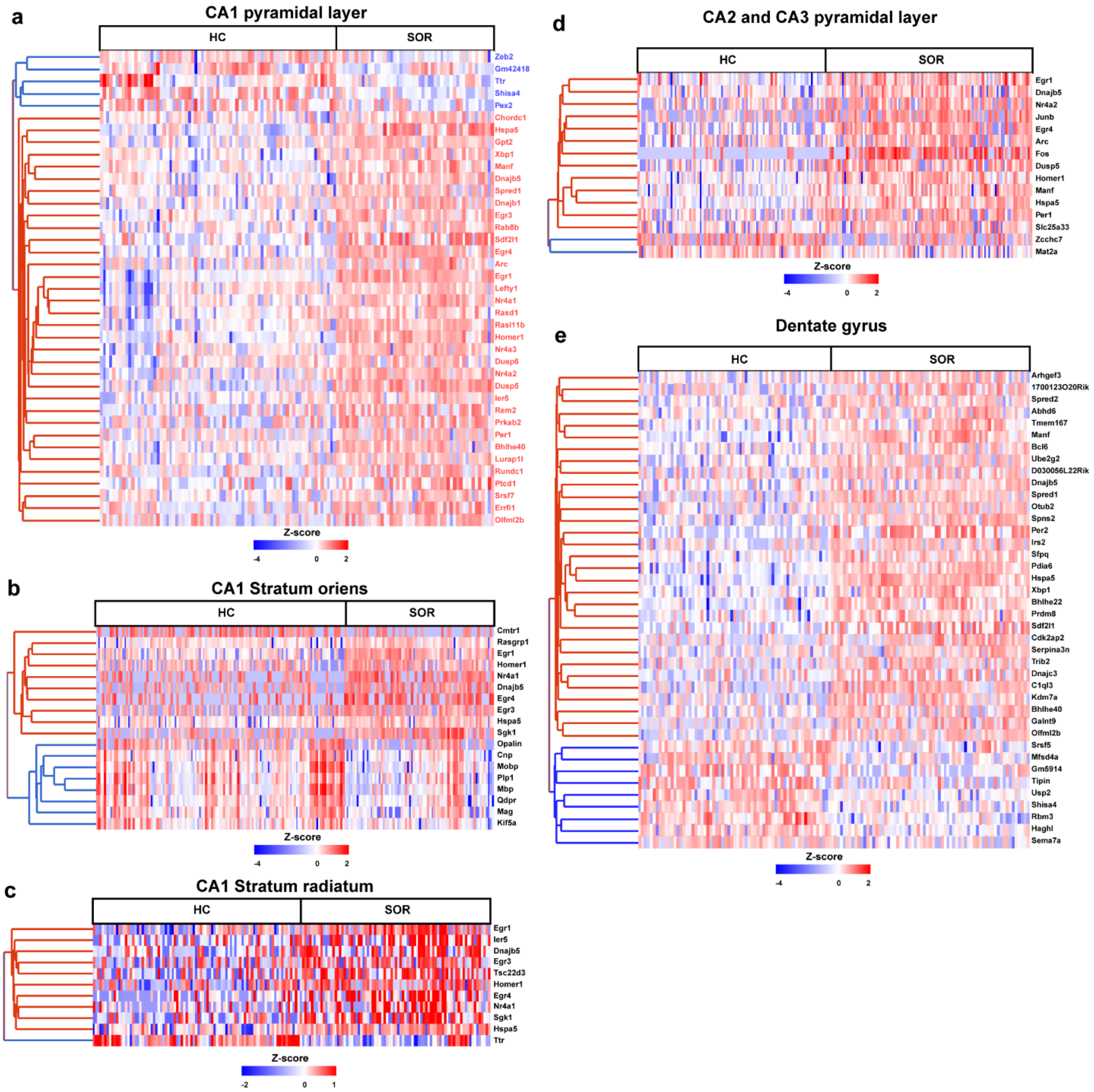

Supplementary Figure 3. Heatmaps showing top significant DEGs from different hippocampal subregions. a, CA1 pyramidal layer. **b,** CA1 stratum oriens. **c,** CA1 stratum radiatum. **d,** CA2 and CA3 pyramidal layers. **e,** Dentate gyrus.

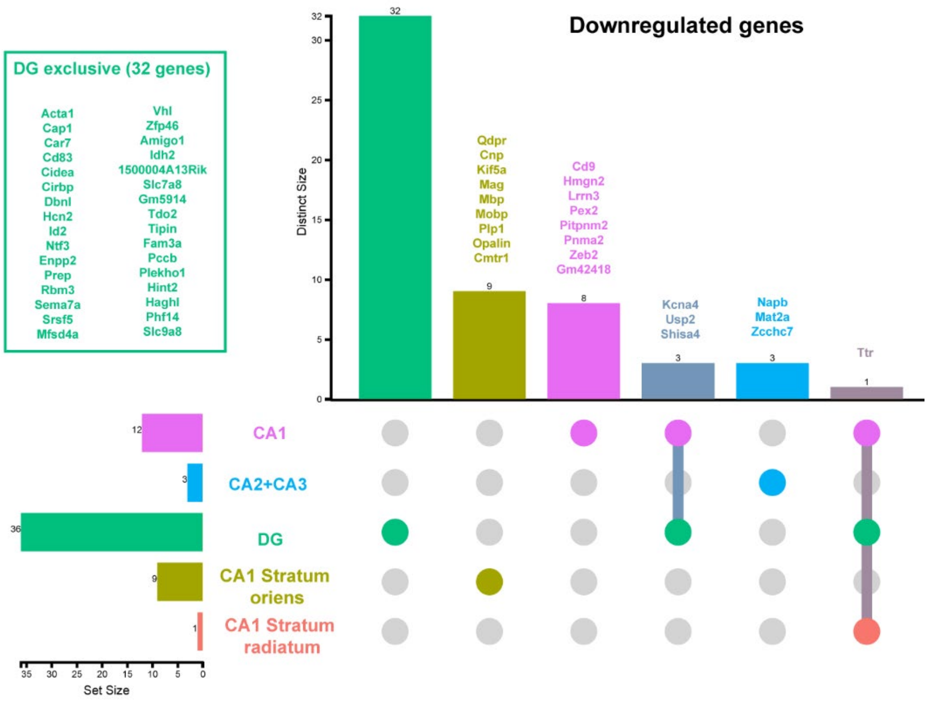

Supplementary Figure 4. UpSet plot depicting the spatial map of all the significantly downregulated genes in the dorsal hippocampal subregions.

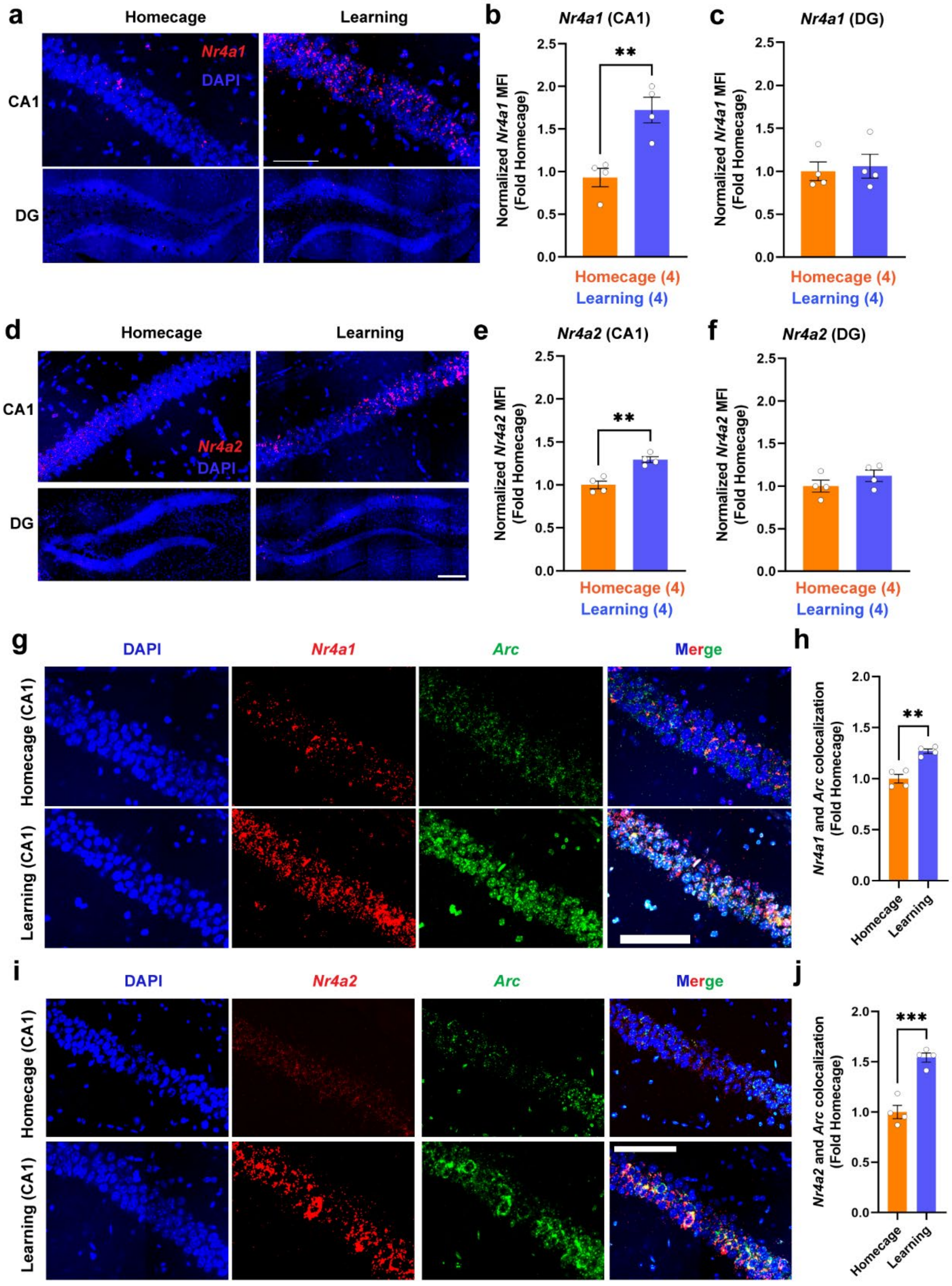

Supplementary Figure 5. Learning-induced expression of Nr4a family members in hippocampal sub-regions using *in situ* hybridization approach. a. Expression of *Nr4a1* in subregions CA1 and DG 1 hr after learning and in home caged animals; Scale bar = 200 µm. **b**. Quantification of a. *Nr4a1* in CA1: Unpaired t-test: t (6) =4.266, p=0.0053. Homecage (n=4), SOR (n=4). Error bars represent ± SEM. **c.** Quantification of a. *Nr4a1* in DG: Unpaired t-test: t (6) =0.3338, p=0.749. Homecage (n=4), SOR (n=4). Error bars represent ± SEM. **d.** Expression of

Nr4a2 in areas CA1 and DG 1 hr after learning and in home caged animals; Scale bar = 200 μm. **e.** Quantification of d. *Nr4a2* in CA1: Unpaired t-test: t (6) =5.311, p= 0.0018. Homecage (n=4), SOR (n=4). Error bars represent ± SEM. **f.** Quantification of d. *Nr4a2* in DG: Unpaired t-test: t (6) =1.258, p= 0.2551. Homecage (n=4), SOR (n=4). Error bars represent ± SEM. **g.** Colocalization of *Nr4a1* and *Arc* in CA1; Scale bar = 100 μm. **h.** Quantification of g. Pearson's coefficient was used to quantify colocalization: Unpaired t-test: t (6) =5.604, p= 0.0014. Homecage (n=4), SOR (n=4). Error bars represent ± SEM. **i.** Colocalization of *Nr4a2* and *Arc* in CA1; Scale bar = 100 μm. **j.** Quantification of i. Pearson's coefficient was used to quantify colocalization: Unpaired t-test: t (6)=6.758, p= 0.0005. Homecage (n=4), SOR (n=4). Error bars represent ± SEM. MFI: Mean Fluorescence Intensity.

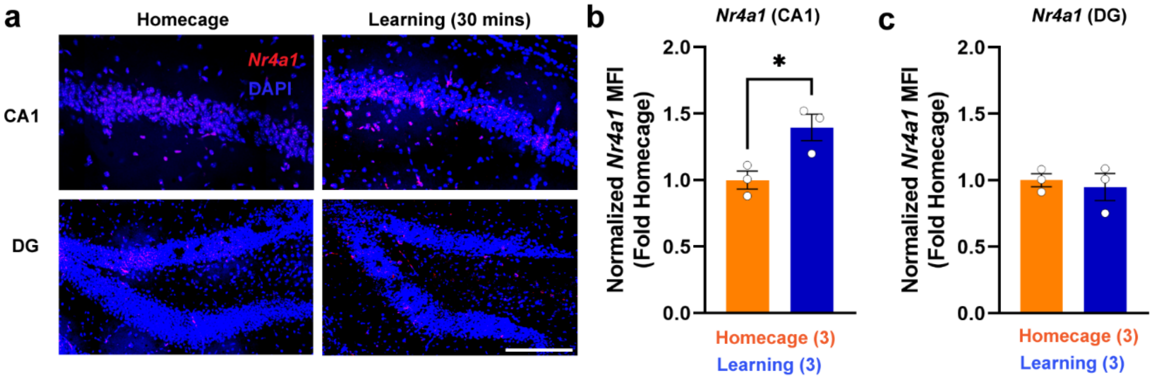

Supplementary Figure 6. Hippocampal subregion-specific expression of Nr4a1 30 mins after learning using *in situ* hybridization approach. a. Expression of *Nr4a1* in CA1 and DG 30 mins after learning and in home caged animals. Scale bar = 200 μm**. b.** Quantification of a. *Nr4a1* in CA1: Unpaired t-test: t (4) =3.304, p= 0.0298. Homecage (n=3), SOR (n=3). Error bars represent ± SEM. c. Quantification of a. *Nr4a1* in DG: Unpaired t-test: t (4) =0.4472, p= 0.6779. Error bars represent ± SEM. Homecage (n=3), SOR (n=3). MFI: Normalized Mean Fluorescence Intensity.

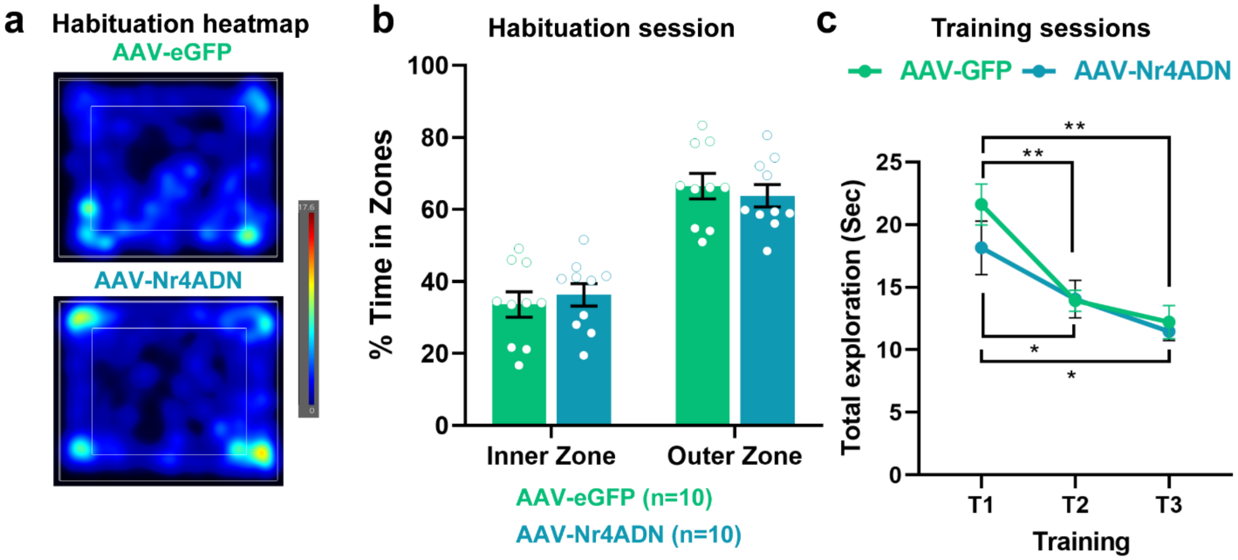

Supplementary Figure 7. Behavioral performance data following AAV-Nr4ADN viral infusion. a. Heatmap showing the location of the mice during the habituation session. **b**. Total time spent in inner and outer zones of the open field during habituation session. Šídák's multiple comparisons tests: Inner Zone (eGFP vs NR4ADN): p=0.7915; Outer Zone (EGFP vs NR4ADN): p=0.7915. Male mice, eGFP (n=10), Nr4ADN (n=10). Error bars represent ± SEM. **c.** Total exploration time for all the objects across training trials. 2way ANOVA: Significant main effect of training trials: F (1.665, 29.97) = 27.05, p<0.0001. Šídák's multiple comparisons test : eGFP: T1 vs. T2 : p=0.0036, T1 vs. T3 : p=0.0041 ; Nr4ADN : T1 vs. T2 : p= 0.0423, T1 vs. T3 : p= 0.0126. Male mice, eGFP (n=10), Nr4ADN (n=10). Error bars represent ± SEM.

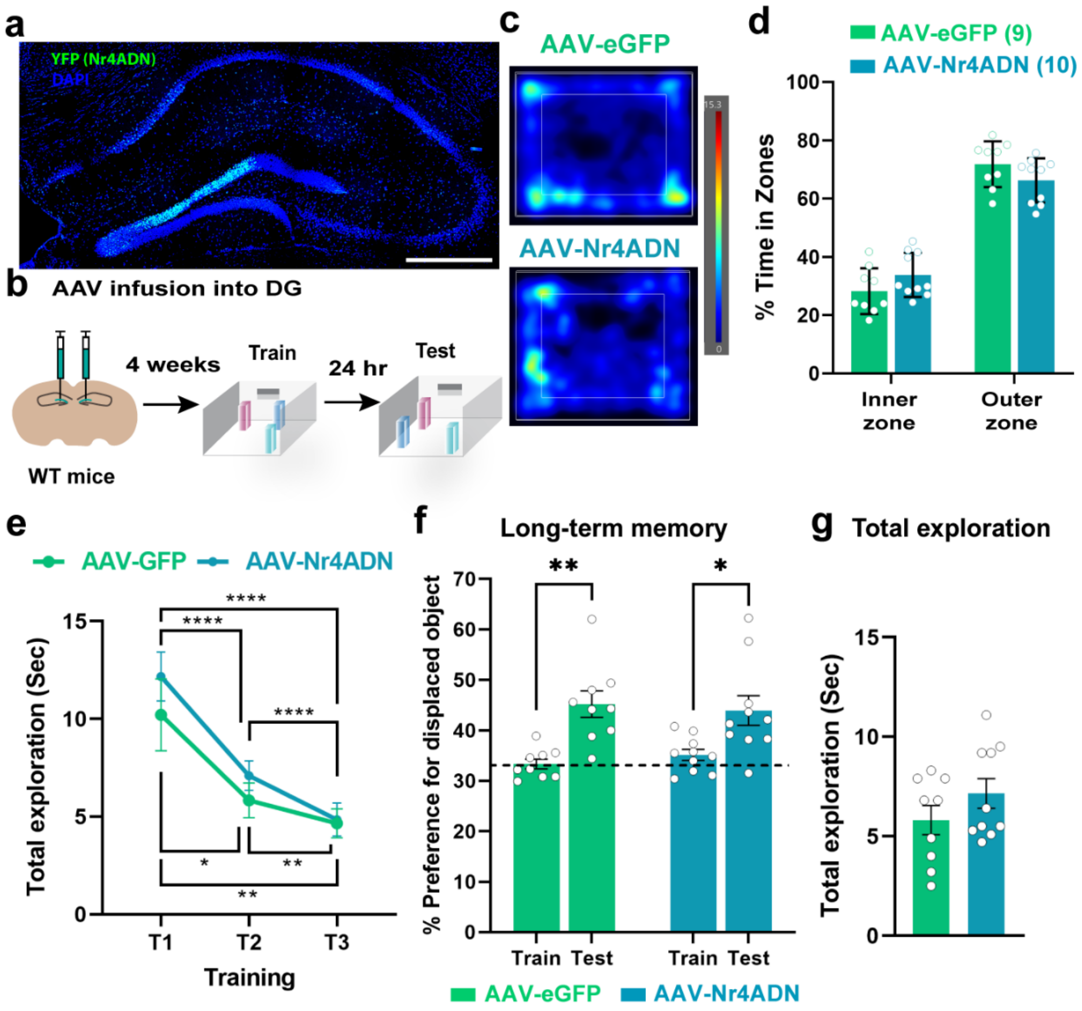

Supplementary Figure 8. Blocking the Nr4a transcription activation function in DG does not impact spatial memory consolidation. a. Immunohistochemistry against YFP to detect the localization and spread of the AAV in the dorsal hippocampus. Scale bar = 500 μm. **b.** Experimental timeline of AAV-infusion into DG followed by spatial learning paradigm. **c.** Representative heatmap showing the location of the mice during the habituation session. **d.** Total time spent in inner and outer zones of the open field during habituation session. Šídák's multiple comparisons tests: Inner Zone (eGFP vs NR4ADN): p=0.255; Outer Zone (EGFP vs NR4ADN): p=0.255. Male mice, eGFP (n=9), Nr4ADN (n=10). Error bars represent ± SEM. **e.** Total exploration time for all the objects across training trials. 2way ANOVA: Significant main effect of training trials: F (1.124, 19.12) = 80.38, p<0.0001. Šídák's multiple comparisons test : eGFP: T1 vs. T2: p= 0.0146, T1 vs. T3: p= 0.0053, T2 vs T3: p= 0.0013; Nr4ADN: T1 vs. T2: p <0.0001, T1 vs. T3: p <0.0001, T2 vs T3: p <0.0001. Error bars represent ± SEM. Male mice, eGFP (n=9), Nr4ADN (n=10). **f.** Long-term memory assessment by evaluating preference for the displaced object (DO) in spatial object recognition (SOR) task. 2way ANOVA: Significant main effect of sessions: F (1, 17) = 18.48, P=0.0005. Šídák's multiple comparisons tests: eGFP (Train vs Test): p=0.0069; Nr4ADN (Train vs Test): p=0.0323. Male mice, eGFP (n=9), Nr4ADN (n=10). Error bars represent ± SEM. **g.** Total exploration time of all the objects during SOR for both the experimental groups. Unpaired t-test: t (17) =1.281, p= 0.2173. Male mice, eGFP (n=9), Nr4ADN (n=10). All the bar and dot plots are mean ± SEM.
