# Supplementary material for: Mapping the spatial transcriptomic signature of the hippocampus during memory consolidation
Source: Nat Commun. 2023 Sep 29;14:6100. doi: 10.1038/s41467-023-41715-7 (PMC10541893; doi:10.1038/s41467-023-41715-7)
Supplement: Supplementary file 3 — Description of Additional Supplementary Files [file 41467_2023_41715_MOESM3_ESM.pdf]

Description of Additional Supplementary Files

File name: **Supplementary Data 1**

Description: Spatial pseudobulk RNA seq dataset generated in this study.

This table presents the results of the differential gene expression analysis conducted on spatial transcriptomic data obtained from the hippocampal region. Pseudobulk data is used, where transcriptomic information from multiple spatial locations within the hippocampus is combined to provide region-level insights. For the Rank-Sum Test Kruskal-Wallis, a non-parametric statistical test, the median gene expression is calculated for each gene across all the spots within the hippocampal subregions. Fold change is calculated as the estimated median expression at the learning condition divided by the estimated median expression at the home cage condition. False Discovery Rate (FDR) correction is then applied to the p-values to account for multiple testing.

File name: **Supplementary Data 2**

Description: Bulk RNA seq dataset generated in this study.

This table displays the differential gene expression result from bulk RNA-seq data obtained from the dorsal hippocampus. The statistical analysis utilized the Generalized Linear Model (GLM) Likelihood Ratio Test. FDR correction was applied to the p-values to control for multiple testing.

File name: **Supplementary Data 3**

Description: Quality metrics of the spatial gene expression and subregion-specific gene lists.

This collection of tables showcases the results of differential gene expression analysis carried out on spatial transcriptomic data across multiple brain regions. Each Excel sheet corresponds to a distinct brain region, and the data within each sheet contains information about genes that display differential expression patterns within that region. For the Rank-Sum Test Kruskal-Wallis applied here, the median gene expression is calculated for each gene across all spots in the tissue section of each brain region. Fold change is calculated as the estimated median expression at the learning condition divided by the estimated median expression at the home cage condition. FDR correction is then applied to the p-values to address multiple testing.
