# Supplementary material for: Mapping the spatial transcriptomic signature of the hippocampus during memory consolidation
Source: Nat Commun. 2023 Sep 29;14:6100. doi: 10.1038/s41467-023-41715-7 (PMC10541893; doi:10.1038/s41467-023-41715-7)
Supplement: Supplementary file 7 — Reporting Summary [file 41467_2023_41715_MOESM7_ESM.pdf]

nature portfolio

Corresponding author(s): Snehajyoti Chatterjee and Ted Abel

Last updated by author(s): Sep 1, 2023

Reporting Summary

Nature Portfolio wishes to improve the reproducibility of the work that we publish. This form provides structure for consistency and transparency in reporting. For further information on Nature Portfolio policies, see our Editorial Policies and the Editorial Policy Checklist.

Statistics

For all statistical analyses, confirm that the following items are present in the figure legend, table legend, main text, or Methods section.

n/a	Confirmed	
☐	☒	The exact sample size (*n*) for each experimental group/condition, given as a discrete number and unit of measurement
☐	☒	A statement on whether measurements were taken from distinct samples or whether the same sample was measured repeatedly
☐	☒	The statistical test(s) used AND whether they are one- or two-sided<br>*Only common tests should be described solely by name; describe more complex techniques in the Methods section.*
☐	☒	A description of all covariates tested
☐	☒	A description of any assumptions or corrections, such as tests of normality and adjustment for multiple comparisons
☐	☒	A full description of the statistical parameters including central tendency (e.g. means) or other basic estimates (e.g. regression coefficient) AND variation (e.g. standard deviation) or associated estimates of uncertainty (e.g. confidence intervals)
☐	☒	For null hypothesis testing, the test statistic (e.g. *F*, *t*, *r*) with confidence intervals, effect sizes, degrees of freedom and *P* value noted<br>*Give P values as exact values whenever suitable.*
☒	☐	For Bayesian analysis, information on the choice of priors and Markov chain Monte Carlo settings
☒	☐	For hierarchical and complex designs, identification of the appropriate level for tests and full reporting of outcomes
☐	☒	Estimates of effect sizes (e.g. Cohen's *d*, Pearson's *r*), indicating how they were calculated

Our web collection on statistics for biologists contains articles on many of the points above.

Software and code

Policy information about availability of computer code

Data collection	Leica CM3050 S cryostat, Olympus BX61 Upright Microscope, Illumina NovaSeq 6000 SP, Odyssey Infrared Imaging System (LI-COR), Olympus FV3000 confocal microscope, QuantStudio 7 Flex Real-Time PCR system.
Data analysis	Visium imaging: Olympus CellSens software v.3.2.<br>Bulk RNA-seq processing: BCBio RNA-sequencing pipeline v1.2.4, STAR aligner v2.6.1, FastQC v.0.11.8, edgeR's quasi-likelihood pipeline, EDASeq v2.30.0, RUVSeq v.1.30.0.<br>Visium data processing: Space Ranger v.1.3.1, STAR v.2.7.10a, Partek Flow package (Build version 10.0.21.0621), Cytoscape 3.9.0 software.<br>SPLIT-seq data processing: split-pipe v0.9.6p, R v4.2.2, Seurat v4.1.0.<br>snATAC-seq data processing: cellranger-atac-2.0.0, Signac v1.7.0, GenomeInfoDb v1.30.1.<br>Western blot: Image Studio Lite ver5.2 (LI-COR)<br>Graphing and statistical analysis: GraphPad Prism v8<br>GitHub repository: https://github.com/YannVRB/Visium-SOR-mouse.git<br>Image analysis: ImageJ

For manuscripts utilizing custom algorithms or software that are central to the research but not yet described in published literature, software must be made available to editors and reviewers. We strongly encourage code deposition in a community repository (e.g. GitHub). See the Nature Portfolio guidelines for submitting code & software for further information.

Data

Policy information about availability of data

All manuscripts must include a data availability statement. This statement should provide the following information, where applicable:

- Accession codes, unique identifiers, or web links for publicly available datasets
- A description of any restrictions on data availability
- For clinical datasets or third party data, please ensure that the statement adheres to our policy

Sequencing files for bulk RNA-seq, spatial transcriptomic, SPLIT-seq and snATAC-seq have been made publicly available through GSE223066.
mm10 reference genome for spatial transcriptomic: https://cf.10xgenomics.com/supp/spatial-exp/refdata-gex-mm10-2020-A.tar.gz
mm10 reference genome for BCBio bulk RNA-seq pipeline: https://github.com/bcbio/bcbio-nextgen/blob/master/config/genomes/mm10-resources.yaml
mm10 reference genome for snATAC-seq: https://cf.10xgenomics.com/supp/cell-atac/refdata-cellranger-arc-mm10-2020-A-2.0.0.tar.gz
mm10 reference genome for split-pipe pipeline: https://ftp.ensembl.org/pub/release-109/fasta/mus_musculus/dna/
Mus_musculus.GRCm39.dna.primary_assembly.fa.gz. https://ftp.ensembl.org/pub/release-109/gtf/mus_musculus/Mus_musculus.GRCm39.109.gtf.gz

Research involving human participants, their data, or biological material

Policy information about studies with human participants or human data. See also policy information about sex, gender (identity/presentation), and sexual orientation and race, ethnicity and racism.

Reporting on sex and gender	N/A
Reporting on race, ethnicity, or other socially relevant groupings	N/A
Population characteristics	N/A
Recruitment	N/A
Ethics oversight	N/A

Note that full information on the approval of the study protocol must also be provided in the manuscript.

Field-specific reporting

Please select the one below that is the best fit for your research. If you are not sure, read the appropriate sections before making your selection.

☒ Life sciences ☐ Behavioural & social sciences ☐ Ecological, evolutionary & environmental sciences

For a reference copy of the document with all sections, see nature.com/documents/nr-reporting-summary-flat.pdf

Life sciences study design

All studies must disclose on these points even when the disclosure is negative.

Sample size	Sample size for each experiment is indicated in the figures and figure legends for each experiment. Sample sizes were estimated based on previous studies in the field (Schurch, N. J. et al. RNA 22, 839-851 (2016), Liu, Y. et al. Bioinformatics 30, 301-304 (2014)). No statistical methods were used to predetermine sample size.
Data exclusions	No animal or sample was excluded from the analysis.
Replication	Spatial memory assessment experiments were replicated at least two times with reproducible results. In addition to our spatial gene expression data from n=4/group, we also integrated our previous spatial transcriptomics dataset from n=3/group (Bahl, E. et al. bioRxiv (2022), GEO GSE201610) to increase statistical power and improve robustness of the results.
Randomization	In each experimental batch, animals were chosen randomly based on their age. No software for randomization were used. Trained mice were cage-mates of the mice in the homecage condition. Similar strategy was also used for AAV-Nr4ADN experiment. Animals were assigned to behavioral chambers randomly to minimize any bias.
Blinding	Discrimination for objects for memory were scored by experimenter blind to the treatment groups. RNA and proteins were extracted by experimenter blind to the treatment groups. Sections for spatial gene expression were prepared blindly.

Reporting for specific materials, systems and methods

We require information from authors about some types of materials, experimental systems and methods used in many studies. Here, indicate whether each material, system or method listed is relevant to your study. If you are not sure if a list item applies to your research, read the appropriate section before selecting a response.

Materials & experimental systems

n/a	Involved in the study	
☐	☒	Antibodies
☒	☐	Eukaryotic cell lines
☒	☐	Palaeontology and archaeology
☐	☒	Animals and other organisms
☒	☐	Clinical data
☒	☐	Dual use research of concern
☒	☐	Plants

Methods

n/a	Involved in the study	
☒	☐	ChIP-seq
☒	☐	Flow cytometry
☒	☐	MRI-based neuroimaging

Antibodies

Antibodies used	pan-HA (1:1000, Cell signaling), YFP (1:1000, Abcam), Actin (1:10,000, ThermoFisher Scientific), anti-rabbit IRDye 800LT (1:5,000, LI-COR), anti-mouse IRDye 680CW (1:5000, LI-COR).
Validation	All the antibodies were previously validated by the manufacturer and have presented in their respective websites.<br><br>pan-HA (https://www.cellsignal.com/products/primary-antibodies/ha-tag-c29f4-rabbit-mab/3724?_requestid=3119885).<br>YFP (https://www.abcam.com/products/primary-antibodies/gfp-antibody-ab290.html).<br>Actin (https://www.thermofisher.com/antibody/product/beta-Actin-Antibody-clone-AC-15-Monoclonal/AM4302).

Animals and other research organisms

Policy information about studies involving animals; ARRIVE guidelines recommended for reporting animal research, and Sex and Gender in Research

Laboratory animals	Male C57BL/6J mice were purchased from Jackson Laboratories. Mice were maintained under pathogen-free conditions with a 12h light/dark cycle, at a temperature of 21-22 C and a relative humidity of 60-70 % in the Animal care facility of University of Iowa. Male C57BL/6J mice of age 2-3 months were used.
Wild animals	The study did not include any wild animals.
Reporting on sex	Only male mice were included.
Field-collected samples	The study did not include any samples collected at the field.
Ethics oversight	All experiments detailed herein complied with the regulations formulated by the Institutional Animal Care and Use Committee (IACUC) of the University of Iowa.

Note that full information on the approval of the study protocol must also be provided in the manuscript.
